# Mediating role of social support between stigma and social alienation in patients with inflammatory bowel disease: a cross-sectional study

**DOI:** 10.3389/fpsyg.2025.1649317

**Published:** 2025-08-13

**Authors:** Guili Xia, Mei-juan Huang, Qing Chen, Yao Pu, Ling Dong, Yiting Zeng, Ling Wang, Yi-ping Chen

**Affiliations:** ^1^Faculty of Medicine, Macau University of Science and Technology, Macau, China; ^2^Department of Gastroenterology, Shenzhen Hospital, Southern Medical University, Shenzhen, Guangdong, China; ^3^Shenzhen Clinical Research Center for Digestive Disease, Shenzhen, Guangdong, China; ^4^Gastroenterology & Hepatology Unit, First Affiliated Hospital of Sun Yat-sen University, Guangzhou, China

**Keywords:** inflammatory bowel disease, stigma, social support, social alienation, cross-sectional study

## Abstract

**Background:**

Stigma is a prevalent issue with well-documented negative consequences, including its association with social alienation. However, this relationship—and the mechanisms underlying it—remain underexplored in individuals with inflammatory bowel disease (IBD). This study aimed to examine the relationships among stigma, social support, and social alienation in IBD patients in China and to determine whether social support mediates the association between stigma and social alienation.

**Methods:**

IBD patients were recruited from two hospitals in China between July 10 and August 19, 2024, using convenience sampling. Data were collected using the Socio-demographic Questionnaire, the Social Impact Scale (SIS), the Social Support Rating Scale (SSRS), and the Generalized Social Alienation Scale (GSAS), which assessed participants’ demographic characteristics, perceived stigma, social support, and social alienation. Descriptive statistics, common method bias tests, analyses of variance, independent-samples t-tests, Pearson correlation analysis, and structural equation modeling were employed to analyze the data.

**Results:**

A total of 504 participants were included, of whom 65.1% were male, with a mean age of 33.97 ± 9.28 years; 76.6% had been living with IBD for 2 years or more. Stigma was significantly negatively correlated with social support (*r* = −0.418, *p* < 0.01) and positively correlated with social alienation (*r* = 0.664, *p* < 0.01). Social support was also significantly negatively correlated with social alienation (*r* = −0.531, *p* < 0.01). Structural equation modeling showed that stigma negatively predicted social support (*β* = −0.487, *p* < 0.001) and positively predicted social alienation (*β* = 0.572, *p* < 0.001), while social support negatively predicted social alienation (*β* = −0.347, *p* < 0.001). Mediation analysis indicated that social support partially mediated the relationship between stigma and social alienation.

**Conclusion:**

Social support partially mediates the relationship between stigma and social alienation in IBD patients. Targeted interventions to reduce stigma and enhance social support may help mitigate social alienation and improve psychosocial outcomes in this population.

## 1 Introduction

Inflammatory bowel disease (IBD) is a group of chronic, non-specific inflammatory disorders of the gastrointestinal tract with unknown etiology, primarily encompassing ulcerative colitis (UC) and Crohn’s disease (CD; Gros and Kaplan, 2023). With rapid urbanization and socioeconomic development, the incidence of IBD in China has surged. In 2021 alone, 168,077 individuals were living with IBD in China, including 24,941 new diagnoses and 5,640 related deaths (Yu et al., 2024). Projections suggest that the number of IBD patients will reach 1.5 million by 2050 (Kaplan, 2015). This expanding population faces substantial psychosocial challenges. IBD predominantly affects young and middle-aged adults and remains incurable. Its characteristic pattern of chronic relapses and remissions has earned it the moniker “green cancer” (Li et al., 2023; Xia et al., 2023). The recurrent nature of the disease and the difficulty in symptom control often lead to psychosocial maladaptation, significantly impairing quality of life (Roberts et al., 2021). Compared to the general population, individuals with IBD experience heightened exposure to stressors (Zhang et al., 2016), with physiological symptoms and mood disturbances forming a mutually reinforcing cycle. Psychosomatic mechanisms—such as alterations in the brain-gut axis, heightened visceral sensitivity, and elevated pro-inflammatory cytokines—can exacerbate somatic symptoms and trigger disease relapse (Black et al., 2020). While research has extensively examined anxiety, depression, and quality of life in IBD, relatively little attention has been given to social support, stigma, and social alienation. Therefore, this study aims to investigate the interrelationships among stigma, social support, and social alienation in Chinese IBD patients and to determine whether social support mediates the relationship between stigma and social alienation.

### 1.1 Stigma

Disease-related stigma refers to the internal experience of being devalued due to one’s illness (Guo et al., 2020). This can manifest as enacted stigma—direct discrimination by others—or as internalized stigma, arising from perceived negative attitudes and societal judgment (Hearn et al., 2020). Although IBD symptoms are generally invisible, patients often face distressing episodes such as sudden urgency or frequent bowel movements, which can disrupt daily activities and social interactions (Lenti et al., 2020). In many cultures, gastrointestinal symptoms are considered unclean and taboo, further intensifying stigma (Defenbaugh, 2013). In Chinese society, the cultural concept of *Mianzi*—social face or reputation—is deeply embedded and influences how individuals perceive and cope with illness. Rooted in Confucian values, *Mianzi* reflects one’s social prestige, earned through fulfilling expected roles. For IBD patients, symptoms like diarrhea or rectal bleeding may be seen as shameful, potentially harming both personal and familial *Mianzi*, thereby intensifying feelings of stigma (Mak et al., 2015). Studies have documented high levels of stigma among IBD patients, with detrimental effects on their quality of life (Luo et al., 2018a; He et al., 2023; Muse et al., 2021). Moreover, stigma contributes to concealment behaviors that undermine medication adherence (Luo et al., 2018a) and poses a barrier to timely psychological care (Wang et al., 2025). Through its influence on the brain-gut axis, stigma may aggravate physical symptoms and elevate the risk of relapse. These findings underscore the urgent need to address stigma in IBD care and research.

### 1.2 Social alienation

Social alienation refers to the psychological distress arising from unmet needs for social connection (Cacioppo et al., 2011). For IBD patients, frequent medical visits and symptom flare-ups can interrupt work or education and restrict participation in social activities. In China’s collectivist culture, the inability to fulfill expected social roles or engage in group life can lead to being perceived as “unsociable,” fostering social alienation. This disconnection not only impairs social functioning but is also strongly associated with depressive symptoms and poor sleep quality (Chen et al., 2022). Given the elevated risk of mood disorders among IBD patients, addressing social alienation is critical for comprehensive, patient-centered care. However, research on social alienation in this population remains limited. One study reported moderate to high levels of alienation among young and middle-aged IBD patients (Liu et al., 2022). Most existing studies focus on cancer (Liang et al., 2022; Fang et al., 2024; Song and Yao, 2024) and stroke patients (Wu et al., 2023). Evidence from these populations indicates a positive correlation between stigma and social alienation (Wang et al., 2022; Wang et al., 2023; Wu et al., 2023; Liu et al., 2025). Yet, this relationship has not been adequately explored in IBD, and the underlying mechanisms and boundary conditions remain unclear. Addressing this research gap may enhance understanding of the psychosocial dynamics of IBD and inform more effective interventions.

### 1.3 Social support

Social support refers to the perceived or actual assistance received from one’s social network (Shinn et al., 1984) and is commonly categorized into objective and subjective components. Objective support encompasses tangible or observable forms of assistance, including direct material help and participation in social networks or group affiliations. In contrast, subjective support involves emotional experiences and the perceived satisfaction of being respected, supported, and understood, reflecting individuals’ internal assessments of their social environment (Xiao, 1994). Among patients with breast cancer, social support is negatively correlated with stigma (Jin et al., 2021). Similarly, research on inflammatory bowel disease (IBD) typically conceptualizes social support as a general protective factor (Fu et al., 2020), with evidence showing a negative association with social alienation (Liu et al., 2022). However, few studies have examined its role as a mediating mechanism, particularly in the unique psychosocial context of IBD. This oversight limits our understanding of how social support may function dynamically—whether by transmitting, buffering, or transforming the impact of stigma on social alienation. Addressing this gap is essential for developing targeted interventions. By testing social support as a mediator, this study aims to clarify its potential role in mitigating the adverse psychosocial effects of stigma in IBD.

### 1.4 Theoretical framework and research hypotheses

**Figure 1:**
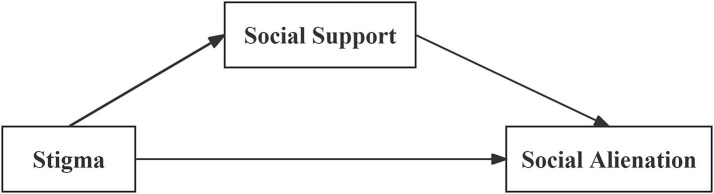
Hypothetical relationship diagram.

According to stress-coping theory, individuals respond to stress through four key components: the stressor, cognitive appraisal, coping strategies, and outcomes (Folkman et al., 1986a; Folkman et al., 1986b). Among these, cognitive appraisal and coping processes are pivotal in determining whether a stressor elicits psychological stress. Within this framework, social support is widely recognized as a stress-buffering external resource (Fu et al., 2023). This study conceptualizes stigma as a chronic stressor that activates cognitive and behavioral responses, depletes coping resources (such as social support), and contributes to adverse outcomes such as social alienation. However, social support can also function as a protective resource, buffering the effects of stigma by alleviating emotional distress, enhancing psychological resilience, and potentially reducing maladaptive behaviors like social withdrawal. Therefore, we hypothesize that social support may positively mediate the relationship between stigma and social alienation. This mediating role may offer new insights for mental health interventions in IBD and inform clinical strategies aimed at psychosocial support. As shown in Figure 1, we developed a theoretical model incorporating the following hypotheses: (1) Stigma is negatively associated with social support and positively associated with social alienation; (2) Social support is negatively associated with social alienation; and (3) social support will partially mediate and attenuate the association between stigma and social alienation.

## 2 Methods

### 2.1 Study design

This study employed a descriptive cross-sectional design to investigate the associations among stigma, social support, and social alienation in patients with IBD in China.

### 2.2 Participant enrollment

Participants were recruited using a convenience sampling method between July 10 and August 19, 2024, from the IBD Center at Shenzhen Hospital of Southern Medical University and the First Affiliated Hospital of Sun Yat-sen University. Eligible patients were identified in the outpatient departments of both tertiary hospitals and invited to participate in the study. Data collection was conducted via structured questionnaires, and responses were compiled into a research database.

Inclusion criteria were: (1) Age ≥18 years; (2) Diagnosis of Crohn’s disease (CD) or ulcerative colitis (UC) based on the 2018 “Consensus on the Diagnosis and Treatment of IBD” by the Inflammatory Bowel Disease Group of the Gastroenterology Section of the Chinese Medical Association (2018); (3) Voluntary participation in the study. Patients who were unable to complete the questionnaires or who had participated in other relevant studies were excluded.

According to Kline (2015), a sample size exceeding 200 is generally recommended for structural equation modeling (SEM). In this study, the sample size was estimated using a widely accepted SEM-specific calculator.^1^ Based on a medium effect size (0.3), power of 0.95, alpha level of 0.01 (Zhang et al., 2023), and a model comprising 3 latent and 11 observed variables, the minimum required sample size was calculated to be 237. To account for a potential 20% attrition rate and sampling error, the target sample size was increased to 296.

### 2.3 Data collection

An electronic questionnaire was developed using a free Chinese online platform^2^ and distributed to IBD patients meeting the inclusion criteria via a link or QR code. Each IP address was permitted a single response. The first page of the questionnaire included an informed consent form, and only participants who provided consent could proceed to complete the survey. A pilot test involving 30 IBD patients in June 2024 established that the minimum completion time was 300 s. Consequently, in the formal survey, responses submitted in less than 300 s were excluded from the analysis.

### 2.4 Measurement

*The socio-demographic questionnaire* was self-administered and aimed to collect the demographic characteristics of IBD patients such as age, gender, educational level.

*The Generalized Social Alienation Scale (GAS)*, Chinese version, originally developed by Jessor and Jessor (1977) and translated and revised by Wu et al. (2015), was used to assess social alienation in IBD patients. The scale comprises 15 items across four dimensions: alienation by others, self-doubt, self-alienation, and meaninglessness. Participants responded using a 4-point Likert scale (1 = “strongly disagree” to 4 = “strongly agree”), with higher scores indicating greater social alienation. The Chinese version of the GAS demonstrated good internal consistency, with a Cronbach’s alpha of 0.81.

*The Social Impact Scale (SIS)*, Chinese version, was employed to assess perceived stigma. Originally developed by Fife and Wright (2000), the SIS was translated by Pan et al. (2007) and revised by Li et al. (2024). The Chinese SIS consists of 22 items across four dimensions: social rejection (9 items), financial insecurity (3 items), internalized shame (4 items), and social isolation (6 items). Responses were recorded on a 4-point Likert scale (1 = “strongly disagree” to 4 = “strongly agree”), with higher scores indicating greater perceived stigma. The total scale demonstrated excellent internal consistency (Cronbach’s *α* = 0.925), with subscale reliabilities ranging from 0.748 to 0.810. Split-half reliability was 0.890, and test–retest reliability was 0.901.

*The Social Support Rating* S*cale* (*SSRS*), developed by Xiao (1994), was used to evaluate levels of social support. Widely validated in China, the SSRS includes 10 items across three dimensions: objective support, subjective support, and utilization of support. Higher scores in each dimension indicate stronger social support (Gu et al., 2018). The total score ranges from 0 to 66, with social support categorized as low (≤20), average (21–30), high (31–40), or very high (>40; Zhang et al., 2021). The SSRS showed strong reliability, with a retest coefficient of 0.92 and item consistency between 0.89 and 0.94 (Xiao, 1994).

### 2.5 Ethical considerations

Ethical approval was granted by the Ethics Committee of Shenzhen Hospital, Southern Medical University (No: NYSZYYEC2024K044R001). All eligible participants were informed about the study’s purpose, procedures, and their rights, including the ability to withdraw at any point during the survey. After providing electronic informed consent, participants completed the questionnaire independently and anonymously. All data were encrypted and stored securely, with participants having the option to request deletion within 30 days of submission.

### 2.6 Data analysis

All statistical analyses were conducted using IBM SPSS 27.0 and AMOS 27.0. (1) Normality of the data was evaluated using the Kolmogorov–Smirnov one-sample test and P–P plots, confirming that the data followed a normal distribution. (2) Common method bias was assessed using Harman’s single-factor test (Podsakoff et al., 2003). Principal component analysis extracted 10 factors with eigenvalues >1; the first factor accounted for 29.40% of the total variance, below the 40% threshold, suggesting no significant common method bias (Podsakoff et al., 2003). (3) Descriptive statistics, one-way ANOVA, and independent sample t-tests were used to examine demographic differences in GAS, SIS, and SSRS scores. (4) Pearson correlation analysis explored relationships among the study variables. (5) A hypothetical mediation model was tested using maximum likelihood estimation in AMOS 27.0. Model fit was evaluated using both absolute and relative indices. Absolute fit indices included χ^2^/df and Root Mean Square Error of Approximation (RMSEA), with values of χ^2^/df < 3 considered a good fit and <5 considered acceptable; RMSEA values <0.05 indicated a good fit, and <0.08 indicated an acceptable fit. Relative fit indices included CFI, GFI, AGFI, IFI, NFI, RFI, and TLI, with values >0.90 interpreted as indicative of a good model fit (Finkelstein, 2005). A bootstrapping method with 5,000 resamples and 95% confidence intervals was used to test mediation effects. A *p*-value <0.05 (two-tailed) was considered statistically significant.

## 3 Results

### 3.1 Sample characteristics

A total of 598 IBD patients were surveyed. Of these, 94 responses were excluded due to completion times under 300 s, resulting in a final sample of 504 valid responses (effective response rate: 84.3%). The majority of participants were male (65.1%), with a mean age of 33.97 ± 9.28 years. Most respondents (76.6%) had been diagnosed with IBD for at least 2 years.

Descriptive statistics, ANOVA, and independent sample *t*-tests were used to analyze the associations between demographic characteristics and GAS, SIS, and SSRS scores (Table 1).

**Table 1:** Associations among socio-demographic characteristics and variable scores (*N* = 504).

Variables	Category	n (%)	Stigma			Social support			Social alienation		
			M ± SD	t/F	*p*	M ± SD	t/F	*p*	M ± SD	t/F	*p*
Gender	Male	328 (65.1)	54.36 ± 10.88	−0.179	0.858	32.86 ± 8.99	−0.886	0.376	37.07 ± 5.21	0.193	0.847
	Female	176 (34.9)	54.55 ± 10.93			33.59 ± 8.44			36.97 ± 5.48		
Age	18–30	212 (42.1)	55.38 ± 10.75	0.965	0.409	29.98 ± 7.56	20.285	<0.001^**^	37.66 ± 5.29	2.066	0.104
	31–40	178 (35.3)	53.87 ± 11.74			34.44 ± 8.68			36.34 ± 5.40		
	41–50	75 (14.9)	53.56 ± 9.88			35.81 ± 8.86			37.08 ± 5.00		
	≥51	39 (7.7)	53.43 ± 9.27			38.85 ± 9.62			36.70 ± 5.23		
Disease duration	<2 years	118 (23.4)	53.23 ± 12.21	−1.258	0.210	34.39 ± 8.71	1.808	0.071	36.99 ± 5.62	−0.099	0.971
	≥2 years	386 (76.6)	54.79 ± 10.44			32.72 ± 8.80			37.05 ± 5.20		
Diagnosis	Ulcerative colitis	135 (26.8)	53.56 ± 11.47	2.009	0.135	34.53 ± 8.81	2.435	0.089	37.31 ± 5.42	2.182	0.114
	Crohn’s disease (active stage)	136 (27.0)	55.99 ± 10.29			32.50 ± 8.90			37.64 ± 5.35		
	Crohn’s disease (remission)	233 (46.2)	54.01 ± 10.83			32.64 ± 8.68			36.52 ± 5.17		
Education level	Associate degree	278 (55.1)	55.86 ± 10.04	5.951	0.003^**^	33.04 ± 8.81	0.182	0.833	37.70 ± 4.99	5.853	0.003^**^
	Bachelor degree	197 (39.1)	52.92 ± 11.57			33.07 ± 8.83			36.39 ± 5.51		
	Master degree or above	29 (5.8)	50.90 ± 12.00			34.07 ± 8.68			35.03 ± 5.91		
Monthly per capita household income (RMB)	≤5,000	231 (45.8)	57.18 ± 10.56	16.596	<0.001^**^	32.39 ± 9.10	2.375	0.094	37.58 ± 5.33	4.869	0.008^**^
	5,001–10,000	187 (37.1)	52.97 ± 10.58			33.23 ± 8.46			37.05 ± 5.05		
	>10,000	86 (17.1)	50.18 ± 10.54			34.79 ± 8.56			35.51 ± 5.50		
Medical payment methods	Medical insurance	472 (93.6)	54.31 ± 10.68	−0.761	0.452	33.28 ± 8.85	1.655	0.099	37.03 ± 5.14	0.002	0.998
	Self-funded	32 (6.4)	56.19 ± 13.71			30.63 ± 7.74			37.03 ± 7.32		

^**^p < 0.01.

### 3.2 Correlations between stigma, social support and social alienation

As presented in Table 2, the mean total scores were 54.43 (SD = 10.89) for stigma (SIS), 33.11 (SD = 8.80) for social support (SSRS), and 35.84 (SD = 6.50) for social alienation (GAS).

**Table 2:** Mean and Standard deviations of stigma, social support and social alienation (*N* = 504).

Variables	Mean ± SD
Stigma	54.43 ± 10.89
Social rejection	20.03 ± 4.64
Financial insecurity	8.58 ± 2.02
Internalized shame	10.89 ± 2.48
Social isolation	14.92 ± 3.66
Social support	33.11 ± 8.80
Objective support	6.81 ± 3.14
Subjective support	20.10 ± 5.53
Utilization of support	6.20 ± 1.91
Social alienation	35.84 ± 6.50
Sense of alienation by others	11.70 ± 2.62
Sense of doubt	9.94 ± 2.06
Sense of self-alienation	7.30 ± 1.74
Sense of meaninglessness	6.90 ± 1.37

Table 3 indicates that stigma was significantly negatively correlated with social support (*r* = −0.418, *p* < 0.01) and positively correlated with social alienation (*r* = 0.664, *p* < 0.01). Additionally, social support was significantly negatively correlated with social alienation (*r* = −0.531, *p* < 0.01).

**Table 3:** Correlation matrix for stigma, social support and social alienation (*N* = 504).

Variables		1	2	3	4	5	6	7	8	9	10	11	12	13	14
1	Social rejection	1													
2	Social isolation	0.785**	1												
3	Internalized shame	0.481**	0.594**	1											
4	Financial insecurity	0.590**	0.656**	0.392**	1										
5	Objective support	−0.285**	−0.264**	−0.231**	−0.236**	1									
6	Subjective support	−0.329**	−0.374**	−0.292**	−0.336**	0.549**	1								
7	Utilization of support	−0.252**	−0.252**	−0.200**	−0.226**	0.429**	0.432**	1							
8	Sense of alienation by others	0.552**	0.599**	0.354**	0.446**	−0.276**	−0.467**	−0.318**	1						
9	Sense of doubt	0.530**	0.605**	0.398**	0.483**	−0.290**	−0.406**	−0.325**	0.715**	1					
10	Sense of self-alienation	0.509**	0.554**	0.396**	0.416**	−0.290**	−0.418**	−0.350**	0.748**	0.650**	1				
11	Sense of meaninglessness	0.335**	0.351**	0.208**	0.295**	−0.253**	−0.424**	−0.344**	0.426**	0.368**	0.417**	1			
12	Stigma	0.910**	0.929**	0.706**	0.747**	−0.307**	−0.395**	−0.280**	0.600**	0.610**	0.571**	0.363**	1		
13	Social support	−0.363**	−0.384**	−0.310**	−0.345**	0.795**	0.918**	0.642**	−0.461**	−0.429**	−0.442**	−0.432**	−0.418**	1	
14	Social alienation	0.597**	0.655**	0.419**	0.506**	−0.334**	−0.518**	−0.397**	0.919**	0.856**	0.862**	0.610**	0.664**	−0.531**	1

^**^p < 0.01.

### 3.3 Mediating role of social support between stigma and social alienation

The model demonstrated a good fit. As shown in Table 4, the modified model yielded satisfactory maximum likelihood estimates. The χ^2^/df ratio was 2.713 (χ^2^ = 111.225, df = 41), and the RMSEA was 0.058. In addition, all incremental and absolute fit indices exceeded 0.900, indicating excellent model fit (CFI = 0.974, GFI = 0.964, AGFI = 0.942, IFI = 0.974, NFI = 0.960, RFI = 0.947, TLI = 0.966).

**Table 4:** Maximum likelihood estimates of the modified model.

Pathway	Standardized coefficient	Standard error	Critical ratio	*p*
Stigma→Social Support	−0.487	0.050	−7.638	<0.001
Social Support→Social Alienation	−0.347	0.110	−6.652	<0.001
Stigma→Social Alienation	0.572	0.084	11.246	<0.001

**Figure 2:**
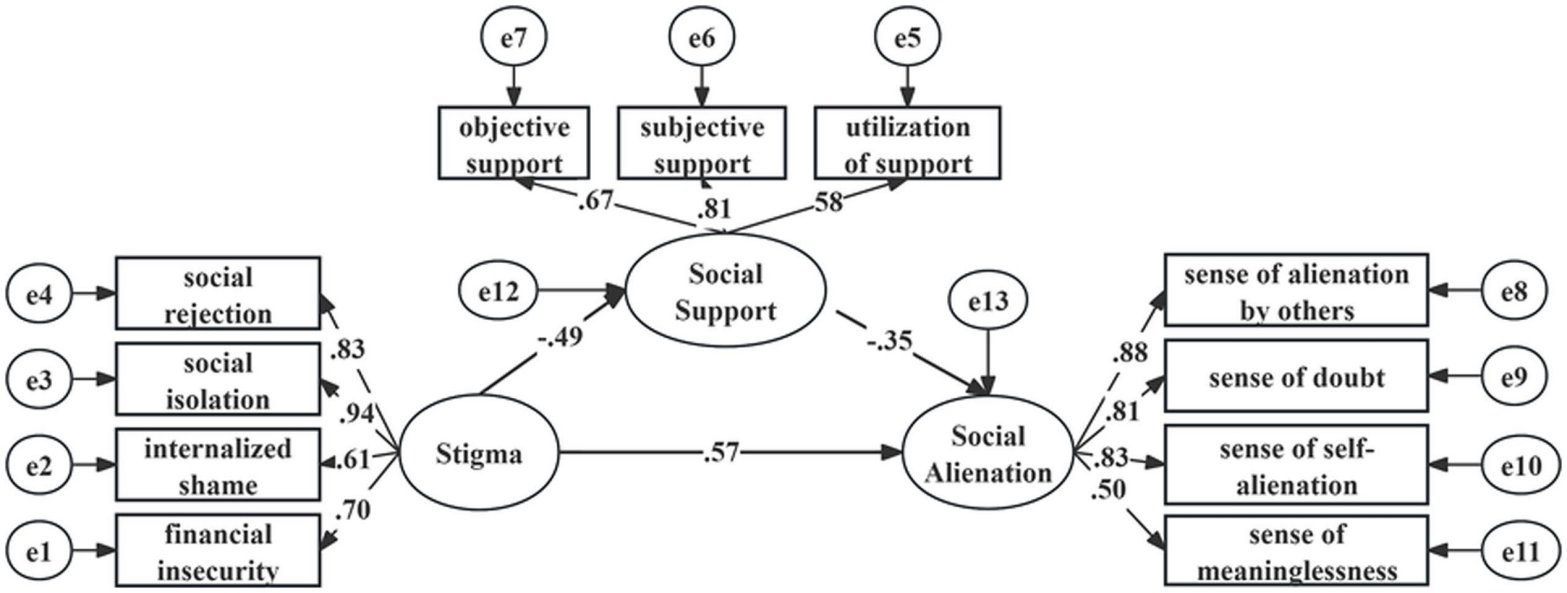
The modified structural equation model.

Figure 2 illustrates the structural relationships between the study variables. Stigma significantly and negatively predicted social support (*β* = −0.487, *p* < 0.001) and positively predicted social alienation (*β* = 0.572, *p* < 0.001). Additionally, social support negatively predicted social alienation (*β* = −0.347, *p* < 0.001). Mediation analysis indicated that social support partially mediated the relationship between stigma and social alienation, with a total indirect effect of 0.169 and a total effect of 0.741.

The mediating effect was further validated using the Bootstrap method with 5,000 replications and a 95% confidence interval (CI). As shown in Table 5, none of the CIs included zero, confirming the statistical significance of the mediation. These results support the proposed model, demonstrating that social support plays a partial mediating role between stigma and social alienation in patients with IBD.

**Table 5:** Bootstrap analysis of path effect significance tests.

Pathway	Estimate	Boot SE	Bootstrap 95% CI	*p*
Standardized indirect effects	0.278	0.049	0.194–0.391	<0.001
Standardized direct effects	0.940	0.089	0.775–1.122	<0.001
Standardized total effects	1.218	0.093	1.051–1.419	<0.001

## 4 Discussion

This study examined the relationships among stigma, social support, and social alienation in Chinese patients with IBD, with particular attention to the mediating role of social support. Findings revealed that stigma was negatively associated with social support and positively associated with social alienation. Social support partially mediated the link between stigma and social alienation. These insights contribute to a deeper understanding of the psychosocial mechanisms affecting IBD patients and offer meaningful implications for both research and clinical practice.

### 4.1 Relationship between stigma, social support, and social alienation

The average stigma score among IBD patients was 54.43 (SD = 10.89), indicating a moderate level of perceived stigma. This score was lower than that reported by He et al. (2023) in patients with CD. The discrepancy may be attributed to differing inclusion criteria: while He et al. focused exclusively on CD patients—who are more vulnerable to psychological distress than those with UC (Petruo et al., 2019)—our study included both CD and UC patients. Furthermore, the mean social support score in this study was 33.11 (SD = 8.80), which was lower than the score reported by Liu et al. (2022) in a sample of middle-aged and young adults with IBD. The mean score for social alienation was 35.84 (SD = 6.50), slightly higher than that reported in Liu’s study. These differences may stem from variations in the age distribution of study participants. Liu’s sample had a higher proportion of middle-aged individuals, who generally have more stable family and social networks, enabling them to better utilize social resources and thereby experience higher social support and lower social alienation compared to younger patients.

The results confirmed that stigma was negatively associated with social support and positively associated with social alienation, supporting Hypothesis 1. This finding aligns with previous research on stroke patients by Wu et al. (2023). Since IBD often manifests during young adulthood—a critical period for career and family development—it can significantly disrupt personal and professional life. The disease not only impairs patients’ functional roles but also increases caregiving and financial burdens on families, contributing to internalized stigma. Elevated stigma levels may discourage patients from seeking external support, reduce social engagement, and ultimately heighten their sense of social alienation, which could adversely affect disease management (Chronister et al., 2013). Therefore, efforts to reduce stigma and mitigate social alienation in IBD patients are of critical importance.

Furthermore, the negative relationship between social support and social alienation supported Hypothesis 2, consistent with Liu et al. (2022) findings. This association may be due to the fact that individuals with higher social support tend to experience greater social integration and community involvement (Wu et al., 2023). As Wang et al. (2017) noted, social support refers to the perceived availability or receipt of assistance from others. It serves as a protective factor against social alienation by offering psychological reassurance and emotional compensation. Social support can positively influence health by optimizing psychological processes such as emotional appraisal and perceived control (Hinzey et al., 2016), thereby enhancing patients’ capacity to cope with illness and reintegrate into society. Consequently, IBD patients with strong social support networks are less likely to experience social alienation.

### 4.2 Mediating role of social support between stigma and social alienation

Finally, the finding that social support partially mediates the impact of stigma on social alienation supported Hypothesis 3 and corroborated the findings of Wu et al. (2023) in stroke patients. According to stress-coping theory, chronic stressors deplete individuals’ coping resources and exacerbate adverse psychosocial outcomes. In this context, stigma functions as a chronic stressor that provokes sustained fears of judgment and rejection. This fear often inhibits self-disclosure and help-seeking behavior, thereby diminishing social support. The resulting lack of support weakens patients’ capacity for social engagement and contributes to heightened social alienation.

Stigma toward individuals with inflammatory bowel disease (IBD)—such as perceptions of “uncleanliness”—can lead to enacted stigma (e.g., workplace exclusion) and internalized shame, both of which diminish patients’ willingness to engage with supportive social networks. Social support serves as a critical mediating factor in disrupting this cycle: it not only alleviates negative emotions but also provides a protective buffer that enables patients to adopt a more constructive perspective on their illness and better accept its impacts (Morey et al., 2021; Zhang and Dong, 2022). When patients perceive adequate support, they are less likely to internalize stigma or withdraw from social interaction. Instead, they are more inclined to seek solutions, reintegrate into social circles, and participate in communal activities, thereby mitigating feelings of alienation. A phenomenological study by Simeone et al. (2023) identified the workplace and social life as the primary contexts in which IBD-related stigma is experienced, often contributing to social isolation. Given the fundamental role of social relationships in well-being, addressing stigma-induced alienation warrants serious attention. Based on our findings, we recommend strategies aimed at both reducing stigma and enhancing social support for individuals with IBD.

Reducing stigma is essential to alleviating social alienation among IBD patients. The sources of stigma are multifactorial. For instance, traditional Confucian values related to “*Mianzi*” (face) may exacerbate stigma, particularly when patients are unable to fulfill familial financial responsibilities or when their condition imposes care and economic burdens on the family (Mak et al., 2015). Such circumstances can strain interpersonal relationships and damage patients’ self-perception. Furthermore, IBD often compromises occupational functioning (Van Der Valk et al., 2014), and patients may experience diminished self-esteem or anger due to derogatory remarks or exclusion by colleagues and supervisors regarding their bowel symptoms or general health (Restall et al., 2016). To counteract these challenges, we recommend the establishment of hospital-based peer support groups, where trained IBD survivors share coping strategies and foster emotional connections, thereby reducing social alienation. Additionally, we advocate incorporating structured discussions on stigma into routine clinical care, led by gastroenterologists and nurses. Increasing public awareness also plays a pivotal role: greater societal knowledge of Crohn’s disease has been associated with reduced stigma (Rohde et al., 2018; He et al., 2023). However, public awareness remains limited (Groshek et al., 2017). While social media offers a fast and accessible platform for information dissemination, individuals frequently engaged in content creation may lack accurate knowledge of IBD, potentially spreading misinformation and exacerbating stigma (Groshek et al., 2017). Therefore, we propose the development of evidence-based, professionally guided online campaigns to educate the public and reduce discrimination and prejudice.

Improving social support is equally critical in reducing stigma-induced social alienation. Social support encompasses three key dimensions: objective support, subjective support, and the degree to which support is utilized. Interventions should therefore aim to both broaden the sources of support and enhance patients’ effective use of available resources. To expand support networks, a comprehensive, patient-centered system is needed. Given the unfamiliar and sensitive nature of IBD symptoms, patients often struggle to disclose their condition—even to close family members (Luo et al., 2018b). As such, support strategies should include strengthening family involvement, organizing regular health education sessions to improve interactions with healthcare providers (Thornicroft et al., 2016), and leveraging digital platforms to create patient associations and peer support communities beyond the hospital setting. Simultaneously, healthcare professionals should work collaboratively with patients’ families to improve the utilization of support. Individual differences in willingness to accept help often hinder the effective use of available resources. Some patients, despite having access to support, may decline assistance, leading to underutilization. Therefore, patients should be encouraged to actively seek help from medical staff, relatives, friends, and colleagues, and to engage in social activities that promote inclusion and reduce alienation.

### 4.3 Limitations

This study has several limitations. First, participants were recruited via convenience sampling from two hospitals in southern China, which may introduce selection bias and limit the generalizability of the results. Future studies should consider broader geographic sampling. Second, although SEM is effective in identifying direct and indirect associations among variables, the absence of longitudinal data limits causal inference. Further longitudinal research is needed to explore the dynamic, long-term effects of stigma on social alienation in IBD populations.

## 5 Conclusion

Our findings demonstrate that social support partially mediates the relationship between stigma and social alienation among Chinese patients with IBD. These results underscore the importance of simultaneously reducing stigma and enhancing social support as dual strategies to alleviate social isolation. We recommend implementing peer-led interventions, clinical training, and public education initiatives to counter the negative effects of stigma and promote social integration.

## Data Availability

The raw data supporting the conclusions of this article will be made available by the authors, without undue reservation.

## Footnotes

^1^ https://www.danielsoper.com/statcalc/calculator.aspx?id=89

^2^ https://www.wjx.cn/
